# Rare Occurrence of a Symptomatic Persistent Sciatic Artery Aneurysm

**DOI:** 10.7759/cureus.31575

**Published:** 2022-11-16

**Authors:** Hasnan M Ijaz, Justin D Mark, Muhammad Lodhi, Waliul Chowdhury, Mustafa Rahim, Rai Kumar

**Affiliations:** 1 Internal Medicine, Hospital Corporation of America (HCA) Florida Westside Hospital, Plantation, USA; 2 Internal Medicine, Dr. Kiran C. Patel College of Allopathic Medicine, Nova Southeastern University, Fort Lauderdale, USA; 3 Internal Medicine, Eastern Idaho Regional Medical Center, Idaho Falls, USA; 4 Internal Medicine, Raleigh General Hospital, Beckley, USA; 5 Vascular Surgery, Beckley Appalachian Regional Hospital, Beckley, USA

**Keywords:** endovascular technique, ir guided embolization, peripheral vascular surgery, vascular surgery, endovascular coiling, persistent sciatic artery aneurysm

## Abstract

Persistent sciatic artery (PSA) aneurysms are a rare cause of gluteal or lower extremity pain. The persistent sciatic artery is an uncommon congenital vasculature anomaly that presents with variable clinical presentation and is prone to cause an aneurysm, thrombosis, rupture, and possible amputation. Thus, early diagnosis is imperative to prevent further complications. We present the case of a 75-year-old female who was diagnosed with a persistent sciatic artery aneurysm after presenting with gluteal and lower extremity pain initially thought to be sciatica. Our patient underwent a successful hybrid open and endovascular approach with a femoral to below-knee popliteal artery bypass and the placement of coils at the proximal and distal ends of the aneurysmal segment.

## Introduction

Persistent sciatic artery (PSA) aneurysm is a rare congenital abnormality with an estimated incidence of only 0.025%-0.06% [1]. The sciatic artery develops in early fetal life but regresses after the development of the femoral artery at 12 weeks of embryonic life. The inferior gluteal artery is a remnant of the PSA. Often presenting with a variable clinical presentation, a PSA aneurysm is known to result in an aneurysm, thrombosis, rupture, and distal embolization. An estimated 46.8% of patients also present with a hypoplastic sciatic artery, while only 7.4% present with an aplastic superficial femoral artery (SFA) [1,2]. 

## Case presentation

**Figure 1:**
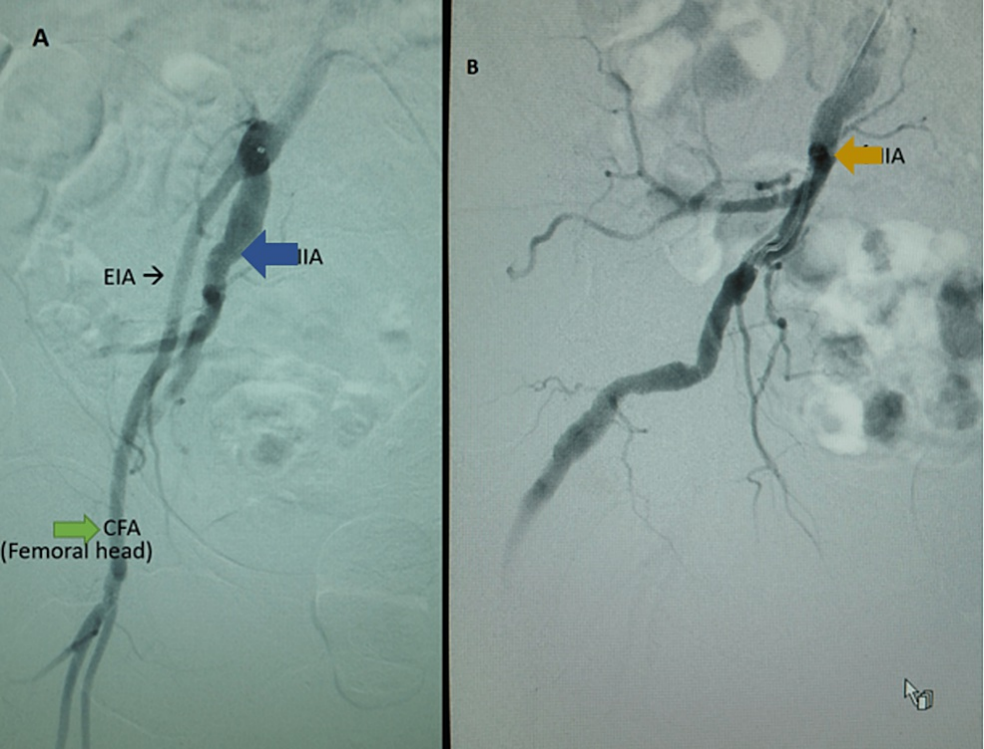
A: External iliac artery (black arrow), internal iliac artery (blue arrow), common femoral artery at the level of the femoral head (green arrow) B: Internal iliac artery (orange arrow)

A 75-year-old female with severe peripheral artery disease (PAD), a history of lower back pain, and sciatica presented with severe right lower extremity claudication. On examination, she had a clinically palpable right femoral pulse, and right proximal and distal Doppler signals were present. The patient was then found to have a pulsatile mass in the right gluteal area. The patient underwent a left transfemoral arteriogram and a selective right lower extremity arteriogram, which showed a persistent sciatic artery with an aneurysm over the ischial tuberosity. The external iliac artery, internal iliac artery, and common iliac artery are shown in Figure 1.

**Figure 2:**
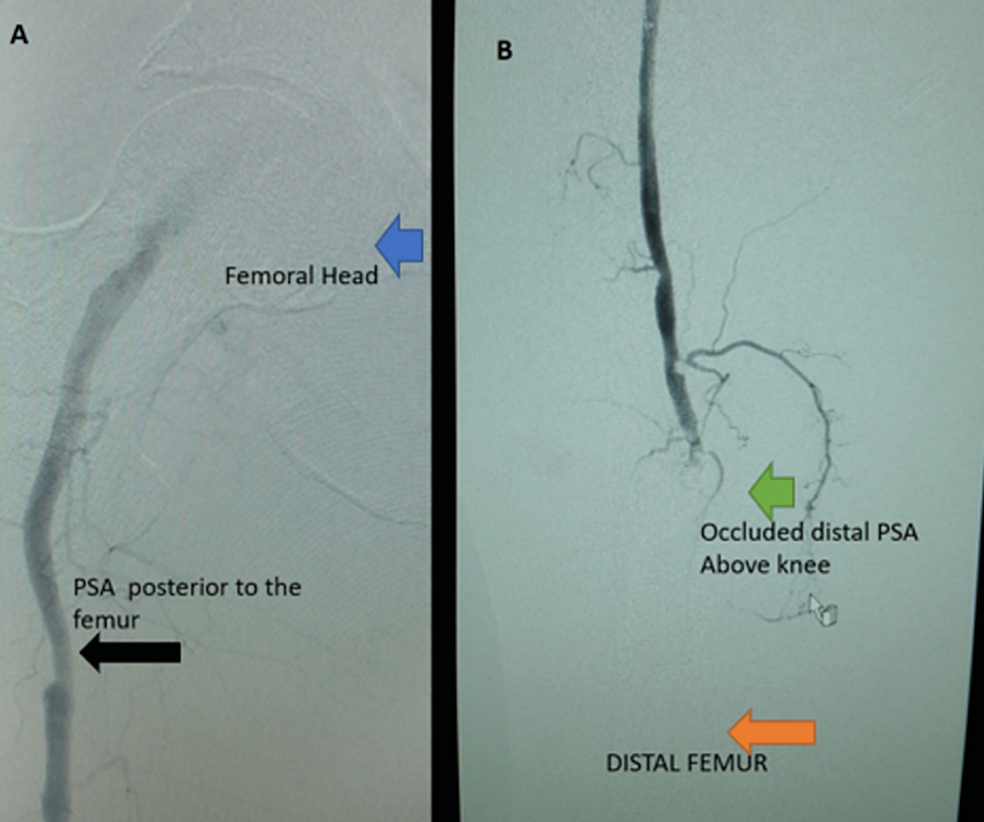
A: Persistent sciatic artery (PSA) posterior to the femur (black arrow), the femoral head (blue arrow) B: Occluded distal PSA above the knee (green arrow), distal femur (orange arrow)

**Figure 3:**
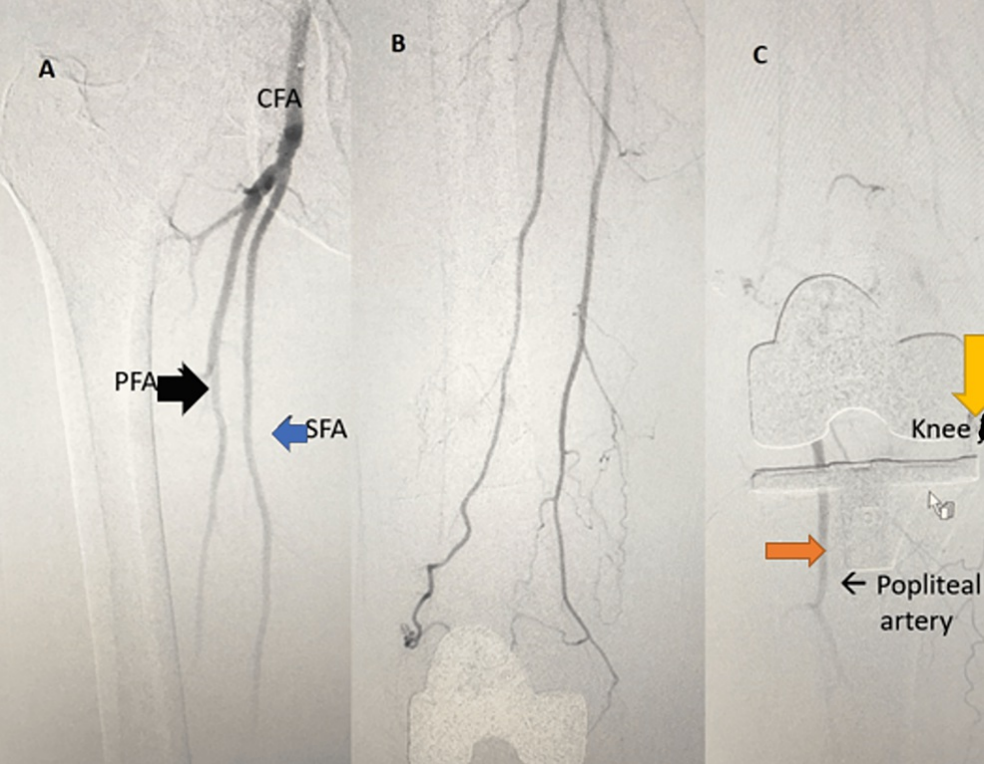
A: Profunda femoris artery (black arrow) B: Superficial femoral artery (blue arrow), C: knee joint (yellow arrow), Popliteal artery (orange arrow)

The PSA was noted to be occluded in the distal thigh with collateral leading to the popliteal artery below the knee with visible tapered distal superficial artery feeding popliteal artery collateral (Figure 2 and Figure 3).

**Figure 4:**
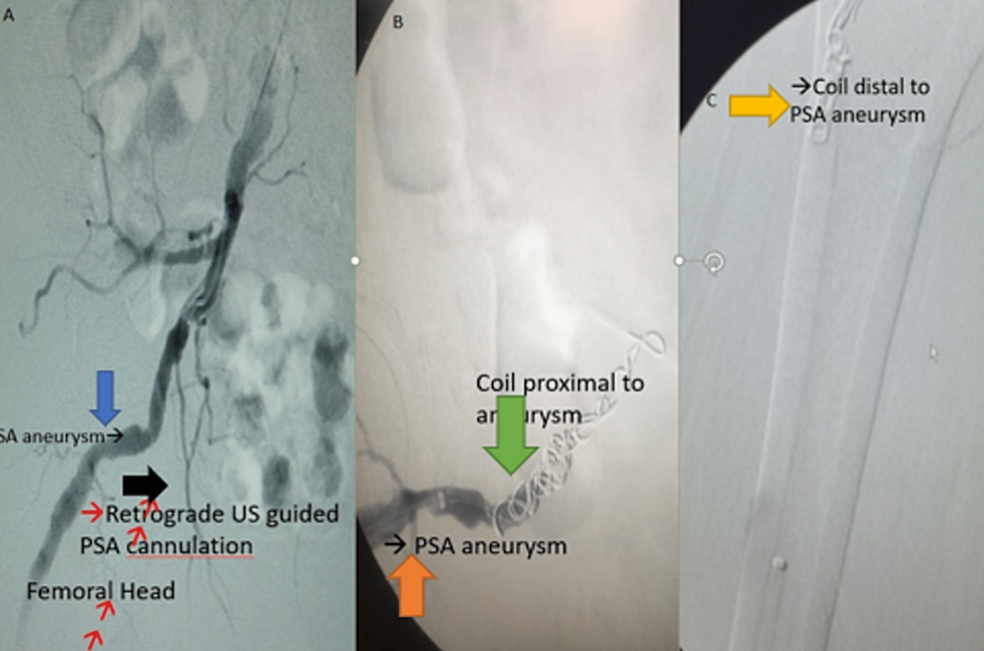
A: Persistent sciatic artery aneurysm (blue arrow), retrograde ultrasound-guided PSA cannulation (black arrow) B: PSA aneurysm (orange arrow); coil proximal to aneurysm (green arrow); C: Distal coil to PSA aneurysm (yellow arrow).

The patient underwent retrograde selective right persistent sciatic artery coil embolization, proximal and distal to the aneurysmal segment, and underwent a right common femoral to below-knee popliteal artery bypass with the greater saphenous vein in situ. Multiple coils were deployed in the proximal sciatic artery just beyond the origin of the internal iliac artery, thus preserving the branches of the internal iliac artery. Proximally, a total of five Nester coils of 16 mm, 14 mm, 14 mm, 7 mm, and 7 mm, respectively, were deployed. The catheter was then retracted, thereby deploying the coils within and distal to the PSA aneurysm (Figure 4). A total of 4 coils of 16 mm, 7 mm, 7 mm, and 7 mm were used distally.

Post-operatively, an arteriogram was performed, which showed early thrombosis of the sciatic artery and preserved lateral branches of the internal iliac artery. At this time, the patient denied any gluteal pain in the area of the aneurysmal sciatic artery, which was initially present before the surgery. The patient remained without further complications and was later discharged without incident. 

## Discussion

**Figure 5:**
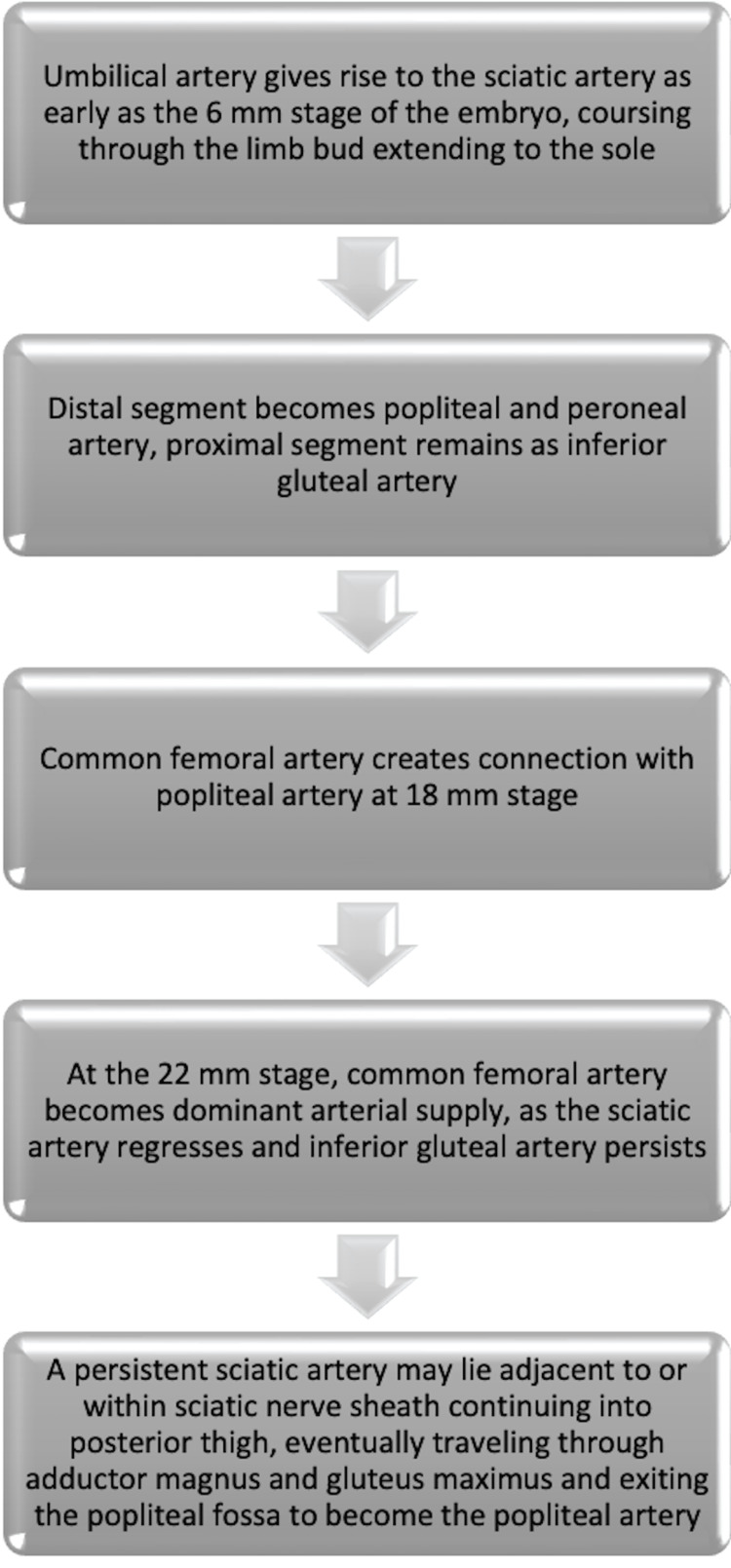
Normal embryological development of the arterial network of the lower extremity; a persistent sciatic artery may be present due to errors in this process

PSA aneurysm is a rare condition previously noted in limited fashion [3-8]. PSA arises due to errors in the embryological formation of the arteries of the distal lower extremity. These are described in Figure 5.

The five classifications of PSA are based on a complete or incomplete presentation and are seen in Table 1. Most PSA aneurysms are complete and require surgical revascularization due to hypoplasia of the superficial femoral artery [3].

**Table 1:** The classification system of PSA as described by Pillet et al. [9], later modified by Gauffre et al. [10]. PFA: persistent sciatic artery; SFA: superficial femoral artery

Type of PSA	Defining Features
Type 1	PSA and SFA are present together.
Type 2a	PSA with incomplete SFA
Type 2b	PSA, while the SFA fails to reach the popliteal artery.
Type 3	An incomplete PSA with the complete development of the SFA.
Type 4	A complete SFA with an incomplete PSA (superior portion).
Type 5a	PSA originates from the median sacral ligament with a fully developed SFA.
Type 5b	PSA originates from the median sacral ligament with an underdeveloped SFA.

The diagnosis of a PSA aneurysm is difficult, as most of the patients remain asymptomatic. Aneurysms, thrombosis, embolism, and radiculopathy are some known complications of PSA aneurysms. Atherosclerosis often damages the arterial wall, with subsequent ballooning and aneurysm formation. Clinical signs of a PSA aneurysm include significant gluteal pain and a pulsatile mass in the buttocks that runs through the sciatic sheath, leading to leg pain due to compression of the sciatic nerve. Acute limb ischemia due to a PSA aneurysm caused by clot embolization from the aneurysm may lead to amputation in an estimated 8% of patients [3-5]. Preferred imaging modalities for diagnosis are Doppler ultrasound and computed tomography angiography (CTA), but magnetic resonance angiography (MRA) may also be used. Doppler ultrasound helps identify the presence of an aneurysm along with the occurrence of thrombosis [4]. Amputation is estimated to occur in 8%-10% of patients following surgical intervention due to thromboembolic complications [11]. After the diagnosis is made, the treatment plan depends on the presenting symptoms, the progression of the aneurysm's length and size, and whether the complete or incomplete variant of the PSA is present. Asymptomatic patients do not require treatment, but they must be monitored regularly due to the risk of aneurysms and thromboembolic complications.

Revascularization of PSA is more beneficial for complete PSA, while the elimination of the aneurysm provides more relief for the incomplete variant. Interventions include endovascular approaches such as coiling, stent grafting, or surgery, which may include open ligation or excision. The endovascular treatment for PSA with stenting is less invasive with a lower risk of stent fracture, migration, perforation, and occlusion with thrombosis after implantation [12-14]. Post-procedure follow-up can be done by annual duplex ultrasonography to exclude a recurrent aneurysm. Depending on the type of PSA, an alternative approach is vascular reconstruction, which is accomplished through open ligation and excision. Revascularization can be accomplished through an interposition graft or bypass from the iliac or common femoral arteries to the distal target [7-10].

## Conclusions

PSA is a rare embryologic anomaly that may lead to complications including aneurysm, thrombosis, and limb loss in severe cases. Early diagnosis and intervention for the symptomatic patient with a PSA aneurysm may prevent these complications. Physicians should consider this diagnosis in a patient who presents with buttock or lower extremity pain and claudication to prevent long-term detrimental effects.
